# Infection with alternate frequencies of SARS-CoV-2 vaccine boosting for patients undergoing antineoplastic cancer treatments

**DOI:** 10.1093/jnci/djad158

**Published:** 2023-08-21

**Authors:** Jeffrey P Townsend, Hayley B Hassler, Brinda Emu, Alex Dornburg

**Affiliations:** Department of Biostatistics, Yale School of Public Health, New Haven, CT, USA; Department of Ecology and Evolutionary Biology, Yale University, New Haven, CT, USA; Department of Biostatistics, Yale School of Public Health, New Haven, CT, USA; Department of Internal Medicine (Infectious Diseases), Yale University, New Haven, CT, USA; Department of Bioinformatics and Genomics, University of North Carolina, Charlotte, NC, USA

## Abstract

Patients undergoing antineoplastic therapies often exhibit reduced immune response to COVID-19 vaccination, necessitating assessment of alternate booster vaccination frequencies. However, data on reinfection risks to guide clinical decision making are limited. Here, we quantified reinfection risks for patients undergoing distinct antineoplastic therapies, given alternative frequencies of boosting with Pfizer-BioNTech BNT162b2. Integrating antibody data following vaccination with long-term antibody data from other coronaviruses in an evolutionary framework, we estimated infection probabilities based on antibody levels and calculated cumulative probabilities of breakthrough infection for alternate booster schedules over 2 years. Annual boosting reduced risks for targeted or hormonal treatments, immunotherapy, and chemotherapy-immunotherapy combinations similarly to the general population. Patients receiving no treatment or chemotherapy exhibited higher risks, suggesting that accelerated vaccination schedules should be considered. Patients treated with rituximab therapy presented the highest infection risk, suggesting that a combination of frequent boosting and additional interventions may be warranted for mitigating SARS-CoV-2 infection.

Effective COVID-19 booster frequencies have been continuously assessed for the general population. However, the potential for reduced immune response to COVID-19 vaccination in patients undergoing antineoplastic therapies leads to a need to evaluate alternative boosting frequencies for this population. Therefore, quantification of the risks of SARS-CoV-2 infection should be conducted and the potential benefits of alternate frequencies of boosting for these patients should be assessed.

We obtained anti-receptor-binding domain antibody levels following Pfizer-BioNTech BNT162b2 vaccination of patients without cancer (1), with cancer undergoing no treatment, or treated with antineoplastic therapeutics: targeted or hormonal treatments, hematopoietic stem cell transplantation, immunotherapies, chemotherapy, immunotherapy and chemotherapy, or rituximab (2). To project antibody waning based on initial responses for each patient cohort in ongoing therapy, we integrated longitudinal anti-N protein and anti-S protein IgG antibody waning data for 6 human-infecting coronaviruses—human coronavirus (HCoV)–OC43, HCoV-NL63, HCoV-229E, SARS-CoV-1, SARS-CoV-2, and Middle Eastern respiratory syndrome (MERS) virus (3)—into an established ancestral and descendent states analysis (3-5), fitting logistic regression models of endemic daily probabilities of infection without additional interventions (3). From ensuing daily probabilities of infection given antibody level, cumulative probabilities of breakthrough infection were calculated (4) for variant-updated booster schedules for members of the general population (5) and for patients with untreated cancer and undergoing continuous treatment over 2 years, scheduled every 1, 3, or 6 months, or 1 or 2 years.

For patients undergoing targeted or hormonal treatments, immunotherapy, a combination of chemotherapy and immunotherapy, or hematopoietic stem-cell transplantation therapy, annual boosting yielded a 2.5-fold reduction in risk over 2 years relative to foregoing boosters (12%–14% vs 29%–31%), similar to noncancer patients (Figure 1). For patients undergoing chemotherapy alone, risks were higher: 18% breakthrough with annual boosting, 8% with 6-month boosting, and 3% with 3-month boosting. For cancer patients receiving no treatment, the risks of breakthrough infection were worse: over 2 years, infection was projected at rates of 22%, 11%, and 6% for yearly, 6-month, or 3-month boosting, respectively. This level of benefit was not achievable for patients undergoing rituximab therapy for which breakthrough infection reached 18% even with monthly boosting. Nearly 2 of 5 rituximab-treated patients were predicted to experience breakthrough infections with annual boosting.

**Figure 1.**
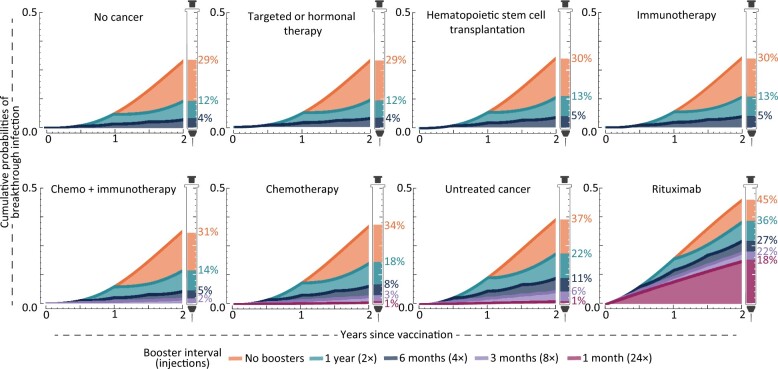
Cumulative probabilities of breakthrough infection with alternate frequencies of updated BNT162b2 booster vaccination following the primary series, for the general population, for patients with no cancer [*n* = 110, Kontopoulou et al. (1)]; and patients undergoing targeted or hormonal therapy, (*n *=* *81); hematopoietic stem cell transplantation (*n *=* *11); rituximab (*n* =* *29); immunotherapy (*n* =* *11); chemotherapy plus immunotherapy (*n* =* *5); chemotherapy (*n* =* *63), from Peeters et al. (2)—and for patients with untreated cancer (*n* = 20), from Debie et al. (6).

Here, we employed postvaccination antibody levels, their waning rates, and associated endemic probabilities of infection to quantify the probability of SARS-CoV-2 infection using alternate booster schedules for patients undergoing antineoplastic therapies. Results for most treatments largely match those for the general population: increased protection with increased frequency of boosting. For patients undergoing chemotherapy alone or with untreated cancers, breakthrough infection risks are elevated and merit consideration for an accelerated vaccination schedule. Moreover, treatment with the B-cell depleting monoclonal antibody rituximab—prescribed for some hematologic malignancies and also as an immune-modulating treatment for other diseases such as rheumatoid arthritis—poses substantially higher short- and long-term risk regardless of booster strategy. Our finding parallels previous findings that patients treated with rituximab are at higher risk for severe COVID-19 (7). Therefore, supplementary interventions such as masking, isolation, and use of prophylactic antibodies targeting SARS-CoV-2 are warranted.

Our study incorporates updating of vaccines to target predominant, emerging strains, as well as the postuptake waning of vaccine efficacy because of antigenic evolution. The resulting infection probabilities are not quantified by early trial data, which have the detriment for long-term prediction that the immune systems of early trial participants are effectively naive to the pathogen and must undergo substantial immunological evolution to produce effective cellular immunity. Instead, our infection probabilities are based on infection data of fully endemic coronaviruses, for which decades of endemic infection data have been collected, analyzed by their evolutionary relation to SARS-CoV-2 so as to produce the best evidence currently available regarding endemic durability of immunity contingent on antibody level. However, there are some notable limitations to our study. Our study does not incorporate the component of antigenic evolution that occurs during the delay between vaccine manufacturing and deployment, which can reduce booster efficacy. Likewise, our results are contingent on future boosters continuing to be updated to match circulating variants with an efficacy level that is minimally as high as currently available boosters. Finally, whether boosting at frequencies that exceed every 6 months would continue to yield consistent antibody elevations targeting consistent antigenic breadth over time for each therapeutic regimen remains unknown and requires additional research.

Regardless, our assessment, based on the best available data regarding an evolving issue at a time when these critical decisions need to be made, provides vital clinical guidance that can aid mitigation of potentially severe SARS-CoV-2 infections in cancer patients undergoing antineoplastic therapies. Further research incorporating antibody responses to vaccines provided during other treatments would facilitate increasingly comprehensive estimation of the benefits of specific schedules of booster vaccination that mitigate risks for vulnerable subsets of the population.

## Data Availability

All data, code, and a detailed readme have been archived on Zenodo and are currently available: doi: 10.5281/zenodo.7963444.
